# Evaluation of aerosol delivery in ventilated patients via vibrating mesh nebuliser versus other delivery devices using a patient-specific in silico whole-lung model

**DOI:** 10.1186/s12931-026-03865-2

**Published:** 2026-08-11

**Authors:** Arash Taki, Barry Murphy, Jakob Richter, Nicolas Schwenck, Maximilian J. Grill, Mary Joyce, Ciaran O Leime, Niamh McNea, Ronan MacLoughlin

**Affiliations:** 1 Aerogen Ltd, Galway, Ireland; 2 Ebenbuild GmbH, Munich, Germany

**Keywords:** In silico, Vibrating mesh nebuliser, Jet nebuliser, Pressurised metered dose inhaler, Digital twin, Mechanical ventilation

## Abstract

**Background:**

In patients requiring mechanical ventilation, normal aerosol dynamics are disrupted by the artificial airway, circuit configuration and humidification systems. These factors can result in substantial drug losses within the ventilator circuit and markedly lower lung deposition than during spontaneous breathing, thereby potentially limiting therapeutic efficacy. A mechanistic proof-of-concept study using an in silico model has been conducted to evaluate aerosol deposition in the lung under differing delivery flow profiles.

**Methods:**

Digital Twinhale^®^ technology was used to generate a subject-specific whole-lung model from CT-derived airway and lung geometry data from a healthy adult, using a 16-generation airway tree and calibrated physiological parameters. Aerosol transport was simulated with three-dimensional Lagrangian particle dynamics using in vitro droplet properties. Phase 1 of the study evaluated Aerogen vibrating mesh nebuliser (A-VMN), jet nebuliser (JN) and pressurised metered dose inhaler (pMDI**)** use under identical delivery flow profiles to assess the impact of the device on delivery efficiency and deposition patterns. In the second phase, deposition via VMN was assessed under two representative simulation scenarios: B1, a sinusoidal (volume controlled ventilation-like) waveform, and B2, a rectangular pressure (pressure controlled ventilation-like) waveform, using matched respiratory settings, including respiratory rate, inhalation-to-exhalation ratio and nominal tidal volume.

**Results:**

Digital modelling allowed observation of differences in device performance, with the A-VMN yielding the highest deposited mass fraction across all lobes and lung regions, as well as the greatest level of functional deposition within the alveolar airways. Modelling ventilator mode-like inspiratory flow profiles allowed the observation that the highest deposited mass fraction was obtained with the B1 flow profile, whereas use of B2 resulted in a greater proportion of the emitted dose being lost to exhalation. Overall, differing flow profile had little effect on central-to-peripheral ratios, indicating that deposition patterns were mainly influenced by aerosol characteristics rather than differences in delivered dose or ventilation parameters.

**Conclusions:**

These findings support the feasibility of using a patient-specific in silico whole-lung model to compare predicted aerosol deposition under controlled conditions. However, further validation in multi-subject models and clinical studies will be required to determine whether these predicted deposition differences translate into meaningful clinical benefit.

**Supplementary Information:**

The online version contains supplementary material available at https://doi.org/10.1186/s12931-026-03865-2.

## Introduction

Aerosol therapy during mechanical ventilation is routinely used by intensivists to deliver medications such as bronchodilators, corticosteroids and antibiotics directly to the lungs [1]. However, achieving effective drug delivery remains challenging as the presence of an artificial airway alters normal airflow dynamics, humidification can lead to aerosol particle growth, and the configuration of the ventilator circuit may promote drug loss through impaction on tubing surfaces [2]. Consequently, lung deposition of aerosolised medications can be substantially lower compared to spontaneous breathing, potentially limiting therapeutic efficacy [3, 4].

Several types of aerosol-generating devices are available for use during mechanical ventilation, each with distinct advantages and limitations. Jet nebulisers (JN) are among the most widely available devices, although their efficiency in ventilated patients is relatively low [2]. The continuous use of compressed gas to generate the aerosol leads to considerable drug loss, particularly during the expiratory phase. Furthermore, temporary disconnection of the ventilator circuit for setup or refilling of JN is required, an open-system process that can result in the loss of positive end-expiratory pressure (PEEP) and increase the risk of ventilator-associated infection [5]. Pressurised metered dose inhalers (pMDI) offer greater delivery efficiency, particularly when actuation is carefully synchronised with the inspiratory phase of mechanical ventilation [2, 3]. Nonetheless, the use of pMDI may still require brief circuit disconnection unless specific in-line adaptors are available, and achieving consistent dose reproducibility can be challenging due to variability in technique and coordination. In contrast, vibrating mesh nebulisers (VMN) represent a newer generation of aerosol delivery technology that produces fine, uniform aerosol particles without introducing additional gas flow, thereby preserving ventilator parameters and patient stability [6]. VMN generally achieve higher lung deposition efficiency than JN and are specifically designed for closed-circuit integration [6, 7]. This design minimises the need for circuit disconnection, reduces the risk of circuit contamination and therefore enhances infection control in mechanically ventilated patients [5, 6].

The mode of mechanical ventilation plays an important role in determining the efficiency of aerosol drug delivery. Volume-controlled ventilation (VCV) commonly uses a constant inspiratory flow that produces a square-wave flow pattern, although other inspiratory flow profiles may also be used. In contrast, pressure-controlled ventilation (PCV) delivers air at a preset inspiratory pressure, resulting in variable tidal volumes and a decelerating flow pattern that gradually decreases as the inspiratory phase progresses [8].

The influence of ventilator mode on aerosol delivery during mechanical ventilation is still not fully understood. VCV and PCV generate different inspiratory flow profiles, which may affect aerosol transport and deposition under specific conditions [8]. Some clinical and experimental studies have reported differences between ventilation modes, including higher deposition using VMN during VCV in selected settings [9, 10]. However, the available evidence remains limited and partly conflicting, and current expert recommendations do not support a proven general effect of ventilator mode on aerosol delivery [11]. For this reason, ventilator mode should be regarded as a potential modifying factor rather than a confirmed determinant of aerosol deposition and further investigation is required.

To date, most comparative evaluations of aerosol delivery systems in the ventilator setting have been conducted under in vitro conditions rather than in clinical trials. Evidence from these laboratory studies have demonstrated the potential performance advantages of VMN compared with other devices, with VMN use achieving significantly higher overall drug delivery, while leaving less than 0.1% of the initial medication volume in the reservoir after nebulisation, compared with 56–63% residual volume observed with JN [12, 13]. These findings underscore the need for further clinical evaluation to validate laboratory results and to establish evidence-based protocols for optimising aerosol therapy in mechanically ventilated patients. However, conducting clinical trials with sufficient statistical power is challenging, costly and time consuming. In order to overcome these limitations, computer-based simulation techniques combining medical imaging, physiological modelling and computational fluid dynamics (CFD), have been developed to replicate airflow patterns and aerosol transport in the lungs. One such advanced software platform, Twinhale^®^, allows a patient-specific digital twin of the lungs to be produced. This can then perform virtual inhalation experiments across cohorts of simulated patients, providing detailed outputs such as local drug deposition profiles. The Twinhale^®^ workflow builds on two relevant validation strands. The aerosol transport and deposition component was validated against independent human in vivo SPECT/CT aerosol deposition data from six healthy subjects and ten inhalation experiments, including two aerosol size distributions and two breathing patterns [14]. Validation endpoints included lobar deposition, central/peripheral (C/P) deposition, and conducting-airway versus alveolar deposition surrogates, with reported correlation coefficients of 0.95 for lobar deposition and 0.98 for C/P deposition. The mechanical component relevant to ventilation was validated in seven mechanically ventilated patients with acute respiratory distress syndrome (ARDS) using CT and ventilatory waveform data, with model-predicted regional ventilation compared against electrical impedance tomography [15]. This study reported 96% correlation for the anteroposterior ventilation profile and an average correlation above 81% across the transverse chest cross-section and dynamic ventilation range. The computational approach combines state-of-the-art modelling techniques for inhalation flow dynamics (CFD) with models of lung tissue mechanics, coupled through fluid–structure interaction (FSI). On top of this integrated framework, Lagrangian particle tracking is applied to accurately represent aerosol behaviour. All simulations are time-resolved rather than averaged, which enhances accuracy and predictive power for deposition outcomes. To ensure feasibility for larger studies, these high-fidelity methods are balanced with reduced-dimensional modelling concepts, enabling reasonable computational effort and making the approach scalable to larger clinical cohorts. In the current study, Twinhale^®^ technology has been used to conduct a mechanistic proof-of-concept, single-subject study, investigating deposition of an active pharmaceutical ingredient (API) delivered via three delivery modalities: VMN, JN and pMDI. In addition, deposition via VMN was assessed under VCV- and PCV-like inspiratory flow profiles to explore the influence of ventilatory parameters on delivery performance.

## Methods

**Fig. 1:**
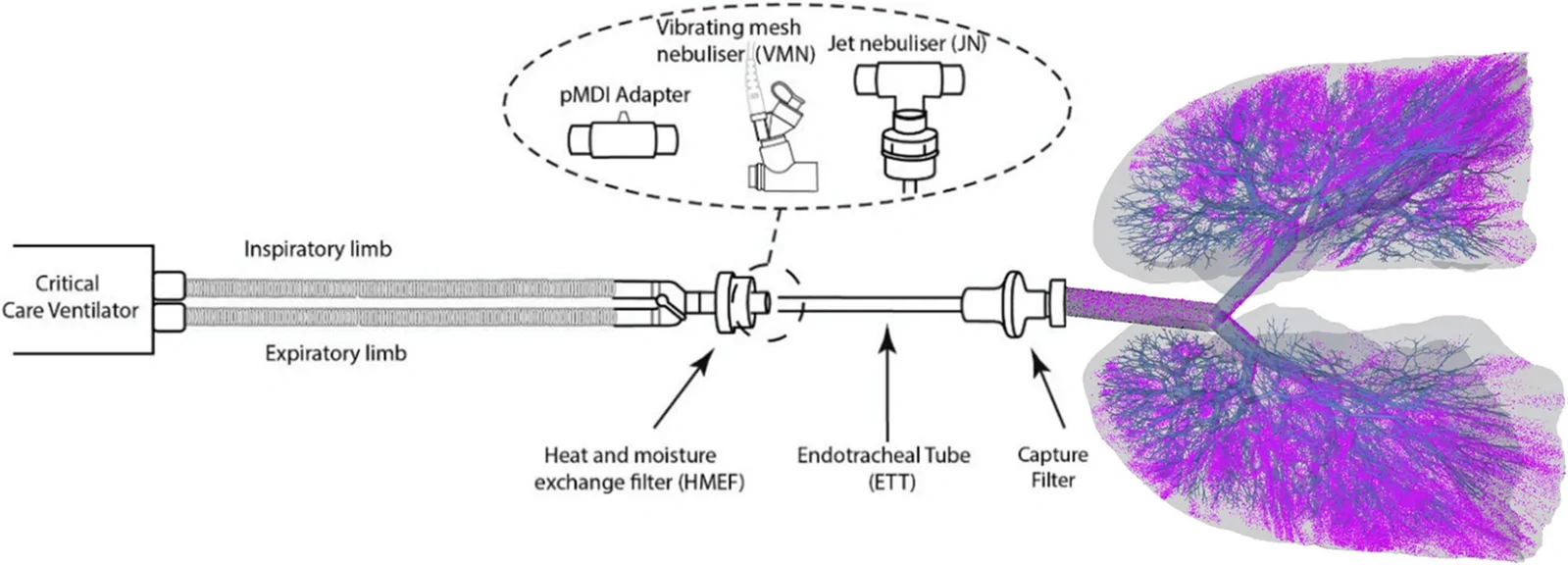
Schematic of ventilation setup and interface to the in silico whole-lung model. ETT, endotracheal tube; HMEF, heat and moisture exchange filter; JN, jet nebuliser; pMDI, pressurised metered dose inhaler; VMN, vibrating mesh nebuliser

A healthy lung geometry from a previously published dataset was selected to derive the subject-specific whole-lung model [16]. Healthy lung data were selected as a validated CT-derived airway dataset with full geometric and physiological characterisation was available, providing a high-quality foundation for the modelling approach [16]. In addition, the primary aim of the study was to evaluate the modelling methodology itself under controlled, reproducible conditions. Data from a single male subject was used, with a functional residual capacity (FRC) of 4170 mL. The ventilation setup and interface of the model is illustrated in Fig. 1.

The process of subject-specific model generation started with the segmentation of airways, lungs and lobes from a high-resolution CT scan. Based on the extracted volumetric information and a physiology-based, space-filing growth algorithm, the full 16-generation tree of conducting airways and alveolar cluster units connected to terminal airways were generated, representing the thoracic respiratory system starting from the trachea down to the distal lung regions. Lagrangian particle tracking was performed from the endotracheal tube (ETT) outlet (tracheal inlet) through the 16-generation airway tree. The ventilator circuit was not modelled computationally. Circuit losses are accounted for by the in vitro delivered dose (DD) values (Supplementary Table 1) applied as the inlet boundary condition. Material parameters of the model were calibrated based on the information from the CT image and the available FRC data. The Twinhale^®^ model is not a conventional full three-dimensional CFD model of the entire lung on a volumetric mesh. Starting from CT-derived lung, lobe, and airway segmentations, a subject-specific full 16-generation conducting airway tree and connected alveolar tissue elements are generated. Airflow and local tissue deformation are computed using a reduced-dimensional whole-lung mechanics model that accounts for bidirectional coupling between airflow and tissue strain. The mechanical material parameters are calibrated from the CT-derived anatomy and available physiological information, including FRC. In the present healthy-lung simulation, no disease-specific regional material heterogeneity was assigned. Aerosol transport and deposition were then simulated using three-dimensional Lagrangian particle dynamics, allowing individual particles to be tracked throughout the conducting airways and alveolar region. Because the whole-lung model uses this reduced-dimensional airflow and tissue-mechanics formulation rather than a conventional volumetric CFD mesh, no conventional turbulence model, CFD mesh size, or mesh-independence study applies to the whole-lung component. Further details of the Twinhale^®^ technology used in this study can be found in the original study [14].

Aerosol physical properties (volumetric median diameter [VMD], geometric standard deviation [GSD] and DD) were characterised in vitro using laser diffraction (Malvern Spraytec, as previously described) [17] at the outlet of the ETT under ventilation conditions matching those used in the simulations (tidal volume, respiratory rate, inspiratory-to-expiratory ratio). This measurement point corresponds to the inlet boundary condition of the computational lung model, ensuring consistency between the in vitro input values and the modelled aerosol entering the lung.

The transport and motion of individual aerosol droplets were simulated using three-dimensional Lagrangian particle dynamics, with individual particles subjected to aerodynamic drag, buoyancy and gravitational forces. Simulations were conducted under the assumption that the subject was in a supine position. Physical properties of the aerosol droplets were based on in vitro measurements.

Scintigraphy-like visualisations were created by projecting the three-dimensional spatial distribution of deposited aerosol mass onto a two-dimensional anterior-posterior plane, analogous to the gamma-camera acquisition geometry used in nuclear medicine scintigraphy. Each pixel in the two-dimensional projection represents the integrated deposited mass of API (salbutamol 2 mg/mL) along the depth axis, normalised to the maximum value in the image. Colour mapping follows a standard heat-map scale from black (zero deposition) through red to yellow/white (maximum deposition), consistent with conventional scintigraphy display. The projection and colour-mapping were performed within the Twinhale^®^ post-processing pipeline.

As this study comprises a single-subject deterministic computational model, inferential statistical comparisons between devices or ventilator modes are not applicable and all results represent exact model outputs for the specific subject and conditions simulated.

### Study phase 1: device comparison

To evaluate the impact of different aerosol-generating devices on pulmonary aerosol deposition, a series of in silico simulations representing Aerogen Solo A-VMN™ powered by the Pro-X controller (Aerogen Ltd, Galway, Ireland), JN (Cirrus2, Intersurgical, UK) and pMDI (Ventolin, GSK, UK) were performed.

A-VMN and JN were operated continuously throughout the breathing cycle; for the pMDI condition, aerosol delivery was performed at the beginning of the inspiratory phase using an in-line pMDI adaptor. As multiple actuations are used clinically and the aerosol plume disperses within the dead-space volume of the ventilator circuit, the aerosol was modelled as a continuous mass emission rate at the computational lung model inlet rather than as a timed bolus. The delivered dose and aerosol size distribution measured at the ETT outlet under the defined bench conditions were used as inlet boundary conditions for the computational lung model. Each was conducted under identical ventilation conditions, using a standardised adult breathing pattern.

The prescribed VCV-like manoeuvre followed a sinusoidal waveform with a tidal volume of 500 mL, a respiratory rate of 15 breaths per minute, and an inspiratory-to-expiratory ratio of 1:1. Distinct aerosol characteristics were incorporated for each device, reflecting in vitro measurements of aerosol size distributions, including VMD and GSD, as summarised in Supplementary Table 1. DD was quantified for each device and expressed as a fraction of the corresponding loaded or metered dose. The analyses focused on quantifying delivery efficiency, defined as the mass fraction of aerosol deposited in the target lung region, and characterising the overall deposition pattern within the lungs.

### Study phase 2: comparison of sinusoidal (B1) and rectangular pressure driven (B2) inspiratory flow profiles under matched ventilation conditions

To investigate the effect of different flow profiles on aerosol deposition, two representative simulation scenarios were performed using identical aerosol characteristics and whilst maintaining consistent respiratory parameters across both cases. B1 (a VCV-like flow profile) was a sinusoidal waveform with a tidal volume of 500 mL; B2 (a PCV-like flow profile) was a rectangular pressure waveform, with a PEEP of 5 cmH_2_O and a pressure control level of 7 cmH₂O above PEEP. The characteristics of the two flow profiles are shown in Supplementary Fig. 1. For both, the breathing rate was fixed at 15 breaths per minute with an inhalation-to-exhalation ratio of 1:1, ensuring that the only difference between the two cases was the shape of the flow profile generated.

DD was defined as the fraction of the loaded or metered dose measured at the outlet of the ETT during in vitro bench testing under representative mechanically ventilated adult circuit conditions. The measured DD and aerosol droplet-size distribution were used as aerosol input boundary conditions for the computational lung model. Analyses focused on delivery efficiency, defined as the mass fraction of aerosol deposited in the target lung region, and on the overall deposition pattern within the lungs. Comparative assessments included evaluation of differences in alveolar deposition, the C/P deposition ratio, and the exhaled aerosol fraction between the two flow profiles. The central and peripheral regions were defined using a concentric-shell approach, consistent with established methodology in pulmonary aerosol deposition research [18, 19]. Each lung volume is subdivided into shells based on radial depth from the lung surface toward the hilum. The inner shells (closer to the hilum and large conducting airways) constitute the central region; the outer shells (toward the parenchyma and pleural surface) constitute the peripheral region. The C/P boundary does not correspond to a fixed airway generation cut-off due to the asymmetric subject-specific airway tree.

## Results

All deposition values are reported as deposited API mass fraction of the loaded or metered dose (%); accordingly, the deposition fractions inherently account for the differences in DD across devices (Supplementary Table 2).

### Device comparison

**Fig. 2:**
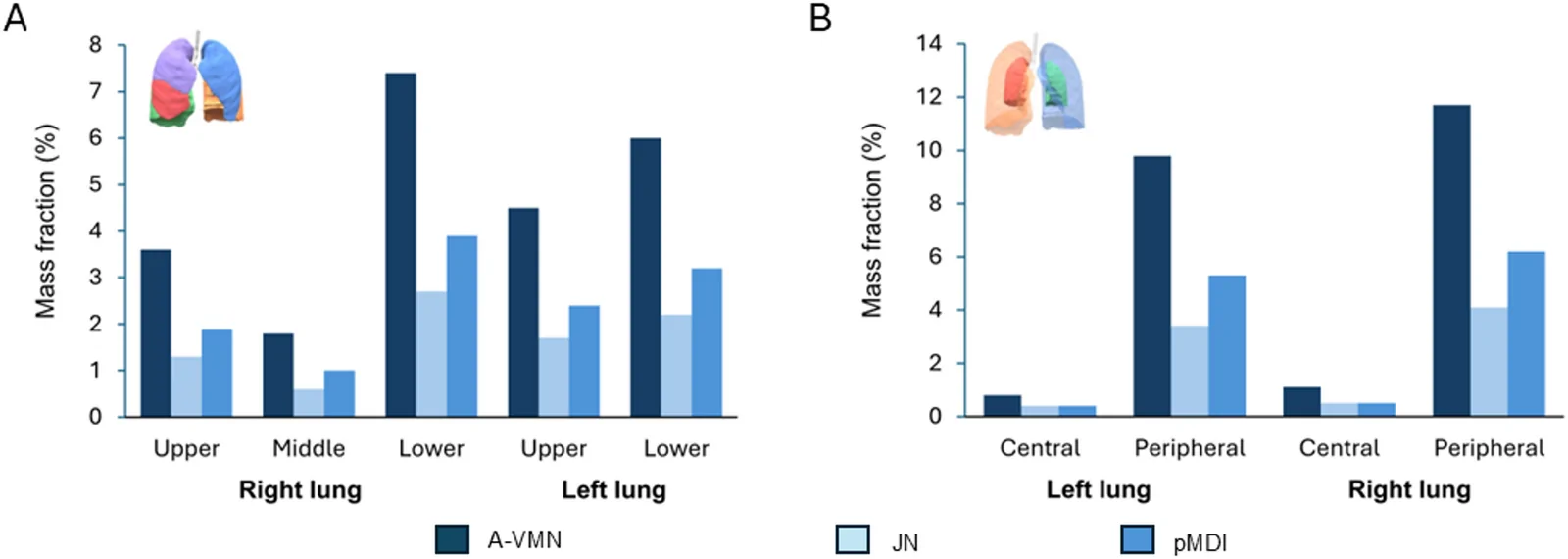
Regional deposition for each device as a fraction of the loaded/metered dose. **A**. Deposition in the lobes of each lung; **B**. Deposition in central and peripheral regions of each lung. A-VMN, Aerogen vibrating mesh nebuliser; JN, jet nebuliser; pMDI, pressurised metered dose inhaler

**Fig. 3:**
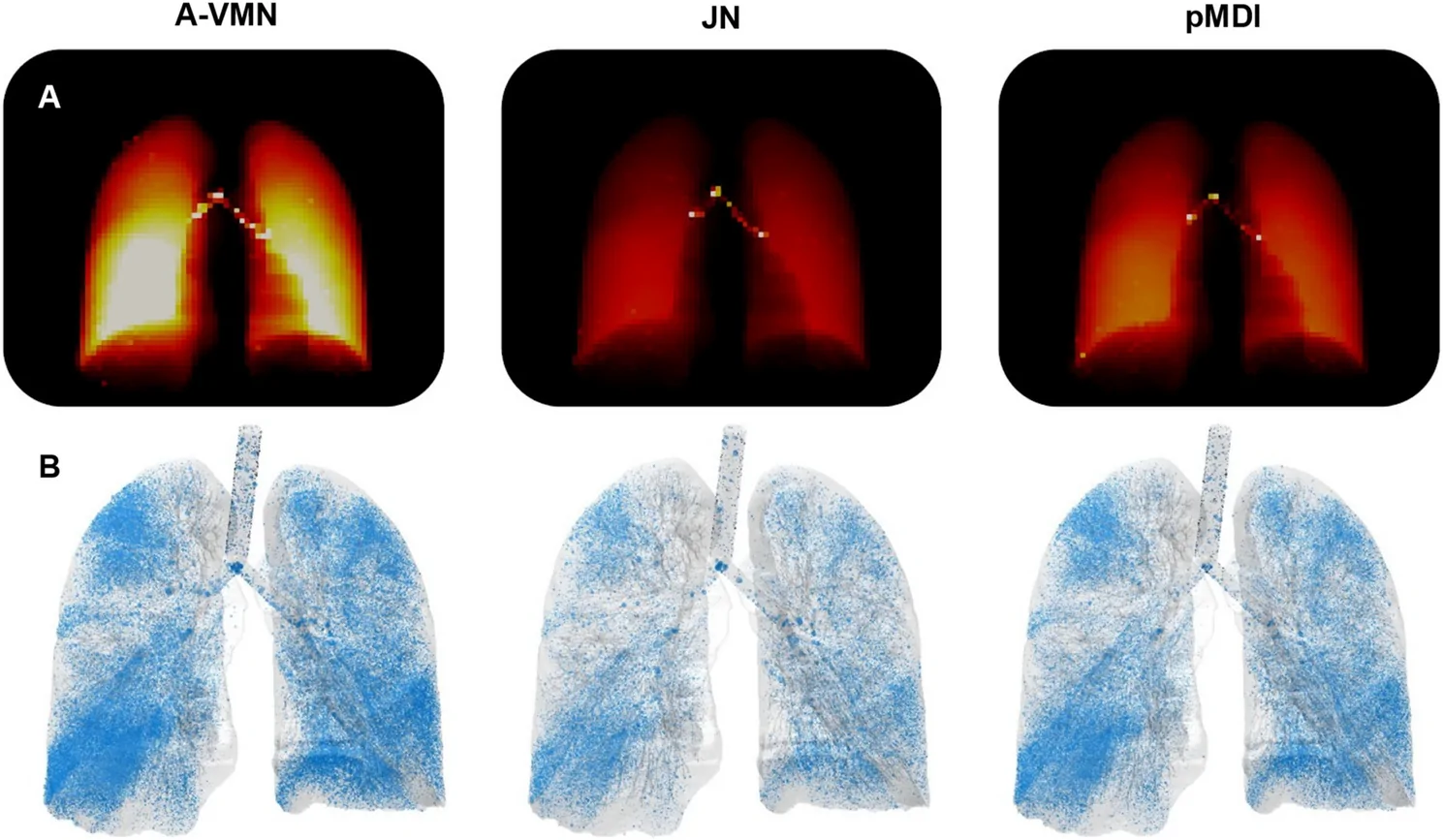
Predicted deposition of active pharmaceutical ingredient for each of the three devices. **A**. Scintigraphy-like visualisation; colour mapping follows a standard heat-map scale from black (zero deposition) through red to yellow/white (maximum deposition); **B**. Three-dimensional rendered images. A-VMN, Aerogen vibrating mesh nebuliser; JN, jet nebuliser; pMDI, pressurised metered dose inhaler

**Fig. 4:**
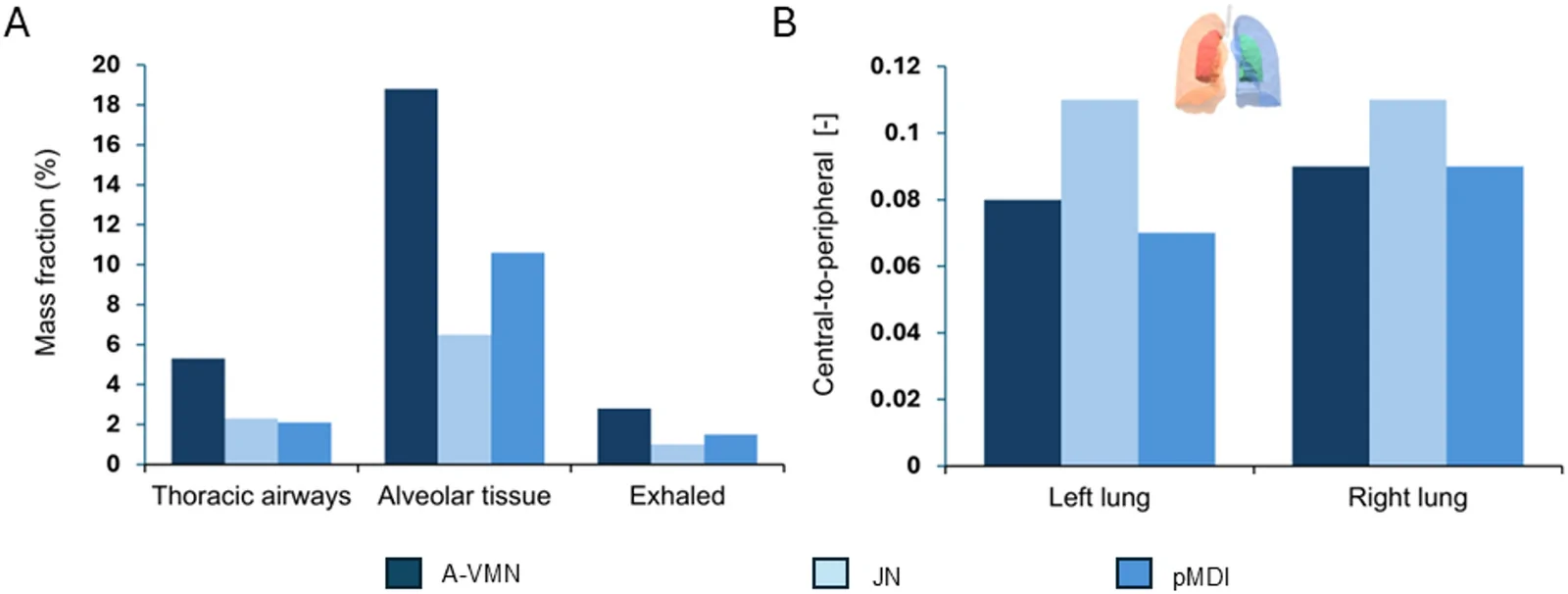
Regional deposition of active pharmaceutical ingredient for each device. **A**. As a fraction of the loaded/metered dose per functional category; **B**. Central-to-peripheral deposition ratio. A-VMN, Aerogen vibrating mesh nebuliser; JN, jet nebuliser; pMDI, pressurised metered dose inhaler

Across all lobes and anatomical regions of the lung, the A-VMN produced the highest deposited API mass fraction (Figs. 2 and 3). Deposition within the trachea and large bronchi, as well as the fraction lost to exhalation, is not shown in these figures. Consistent with the regional findings, functional deposition within the alveolar compartment was also greatest for the A-VMN (18.8% versus 6.5% and 10.6% with JN or pMDI, respectively; Fig. 4A). The proportion of aerosol remaining in the system at the end of the breathing cycle was minimal for all devices, accounting for < 1% of the metered or loaded dose.

The C/P ratio of deposited API mass was comparable between the A-VMN and pMDI, and only marginally lower than that of the JN (Fig. 4B). Differences in DD between devices do not influence the interpretation of C/P ratios, as the DD affects central and peripheral deposition in parallel, cancelling out within each case. Under identical ventilatory flow conditions, the dominant determinant of the C/P ratio is therefore the droplet-size distribution generated by each device. The C/P ratio is governed primarily by the droplet-size distribution (VMD and GSD) rather than by total DD; as both simulations used the same particle input, the similar C/P ratios across modes are expected.

### Comparison of B1 and B2 flow profiles

**Fig. 5:**
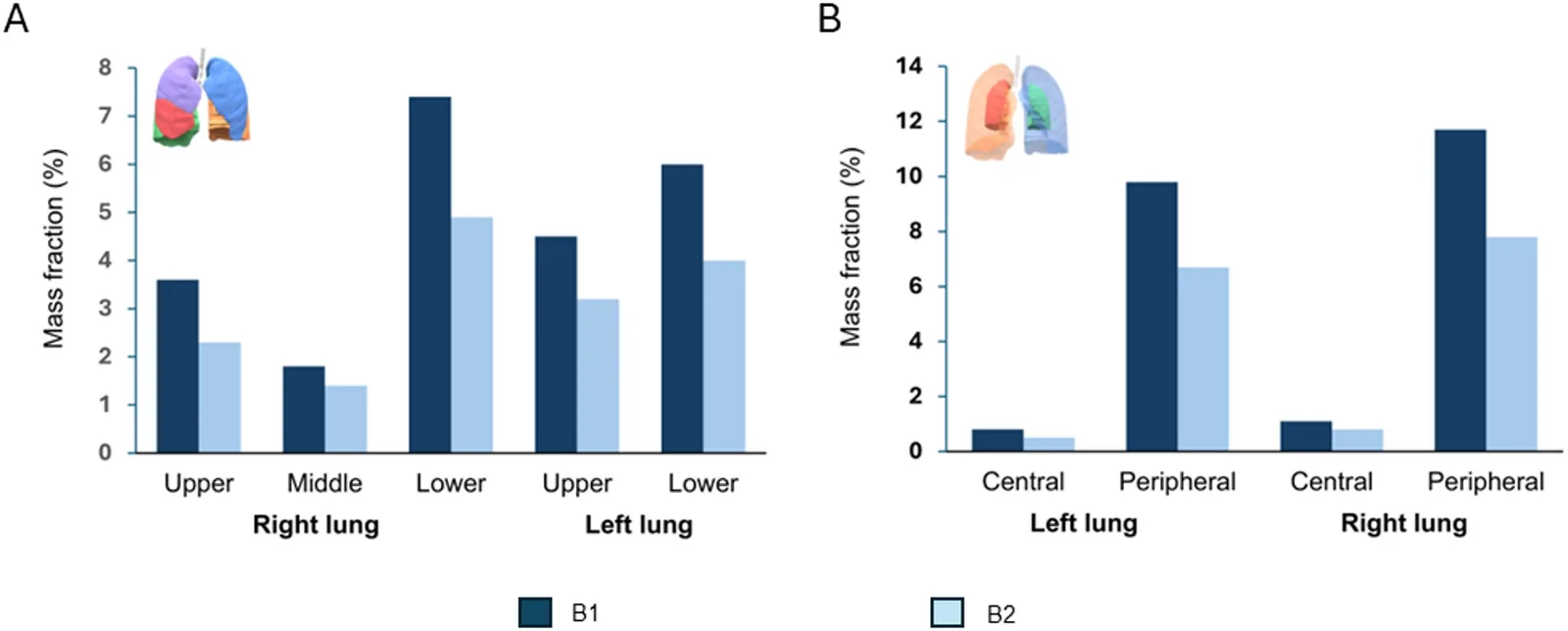
Deposited active pharmaceutical ingredient as a fraction of the loaded dose for the B1 and B2 flow waveforms. **A**. Deposition in the lobes of each lung; **B**. Deposition in central and peripheral regions of each lung. B1, sinusoidal flow waveform; B2, rectangular pressure-driven flow waveform

**Fig. 6:**
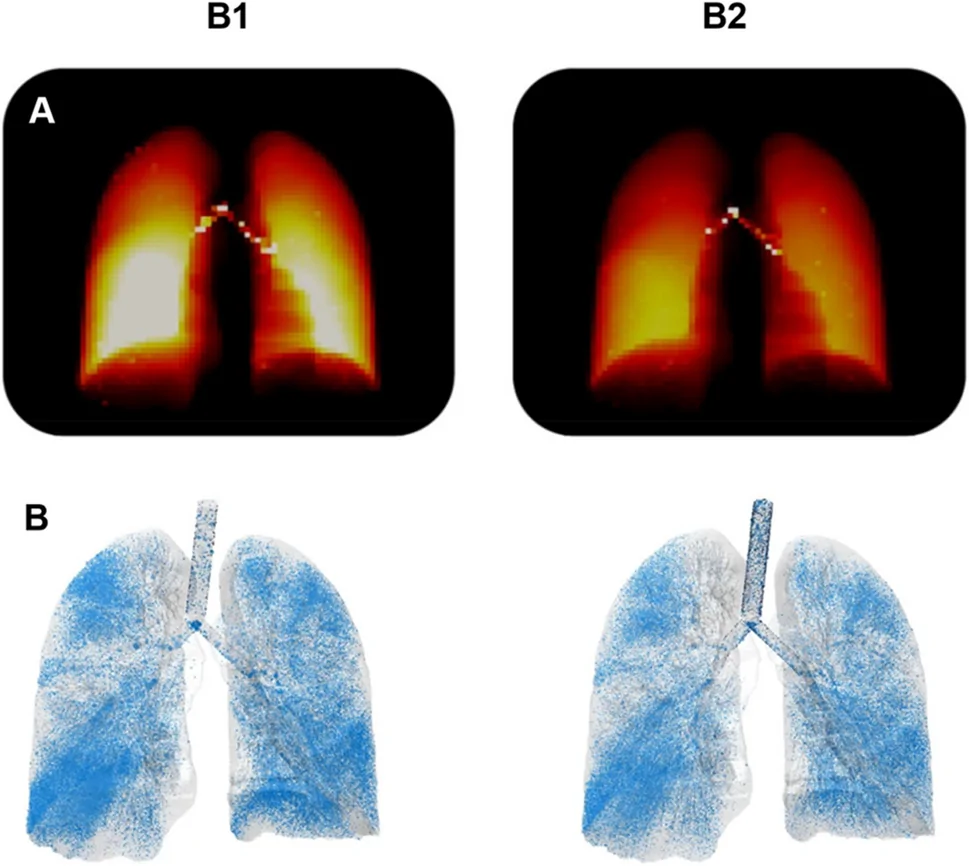
Predicted deposition of active pharmaceutical ingredient for the B1 and B2 flow waveforms. **A**. Scintigraphy-like visualisation; colour mapping follows a standard heat-map scale from black (zero deposition) through red to yellow/white (maximum deposition); **B**. Three-dimensional rendered images. B1, sinusoidal flow waveform; B2, rectangular pressure-driven flow waveform

**Fig. 7:**
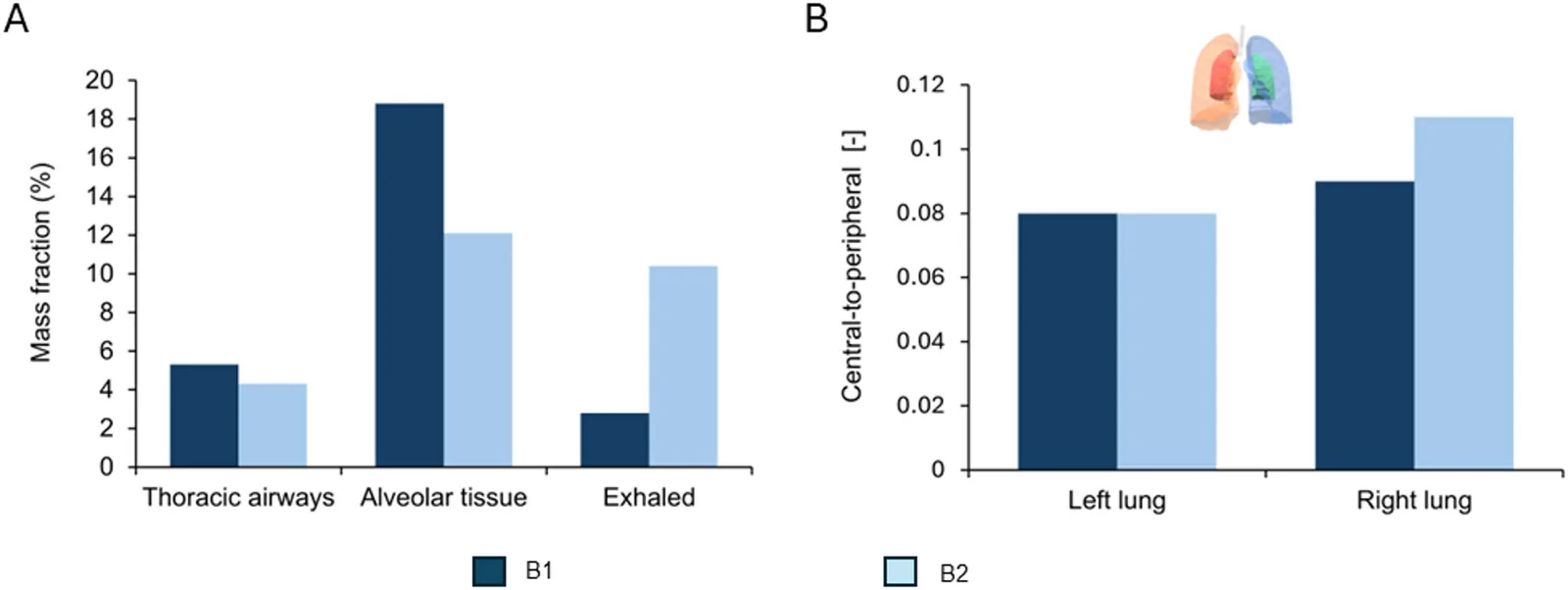
Regional deposition of active pharmaceutical ingredient for the B1 and B2 waveforms. **A**. As a fraction of the loaded/metered dose per functional category; **B**. Central-to-peripheral deposition ratio. B1, sinusoidal flow waveform; B2, rectangular pressure-driven flow waveform

Across the simulated conditions, the greatest fraction of deposited API mass was observed during ventilation with B1, as shown in Figs. 5 and 6. When deposition was evaluated by functional lung region, a higher fraction of the aerosol reached the alveolar compartment during B1, whereas B2 resulted in a greater proportion of the emitted dose being lost to exhalation (Fig. 7A).

Despite the differences in total deposited mass, the C/P deposition ratios were broadly similar between the two flow profiles (Fig. 7B). This was expected, as both simulations used an identical droplet-size distribution. For the left lung, the C/P ratio was 0.08 for both B1 and B2; corresponding values in the right lung were 0.09 for B1 and 0.10 for B2, indicating only minimal variation between modes.

## Discussion

This study presents a novel, high-resolution, single-subject in silico simulation approach designed to compare aerosol deposition across differing aerosol delivery device types and inspiratory flow profiles. It represents the first in silico evaluation incorporating three device scenarios, alongside two flow profiles. Conducted as a single-patient proof-of-concept analysis, the simulations utilised an in silico model to examine regional and lobar differences in aerosol deposition under standardised conditions. The model allowed observation of differences in device performance, with the A-VMN yielding the highest deposited mass fraction across all lobes and lung regions, as well as the greatest level of functional deposition within the alveolar airways. Modelling ventilator mode-like inspiratory flow profiles allowed the observation that the highest deposited API mass fraction was obtained with B1, a VCV-like flow profile.

The efficiency of the A-VMN use observed in the present simulation is consistent with findings from previous imaging-based studies. In a study of ten healthy volunteers, VMN delivered approximately twice the amount of radiolabelled aerosolised drug into the respiratory tract as the JN under comparable conditions [20]. Similarly, evaluation of the pulmonary deposition of technetium-99 m-labelled diethylenetriaminepentaacetic acid in six healthy individuals found that VMN achieved a higher degree of deposition efficiency than JN, although the percentage deposition assessed by SPECT-CT analysis was low in both groups (VMN 3.6% versus JN 1.0%; *p* < 0.05) [21]. The higher DD associated with VMN choice results from a combination of factors, such as a lower residual volume remaining un-nebulised (typically less than 5%) and a lower velocity aerosol that is entrained in the inspiratory flow more efficiently without the losses noted for higher velocity JN and pMDI. The ballistic fraction of the aerosol plume generated by both JN and pMDI tends to impact on connector walls prior to entrainment in the inspiratory flow. The cumulative evidence and experience with VMN devices have ultimately contributed to inclusion in expert recommendations and widespread adoption as the device of choice in clinical trials [11, 22].

In the current study, the C/P ratios for the A-VMN and pMDI were seen to be comparable, while the JN showed a marginally lower C/P ratio. Although the droplet size values are similar across devices, the width of the droplet-size distribution (GSD) differs between the A-VMN and JN. A broader GSD produces a greater proportion of both very fine droplets (which penetrate to the periphery) and larger droplets (which impact centrally), potentially shifting the C/P balance. In addition, differences in aerosol velocity and delivery timing relative to the inspiratory waveform may influence the proximal-to-distal distribution. As this is a single-subject deterministic model, caution is required in drawing mechanistic conclusions and the marginal C/P difference observed may not be clinically meaningful.

Evaluation of differing flow profiles in the current study showed improvement in deposition using B1 (versus B2). This is consistent with previous observations in an in vitro model, where aerosolised iloprost delivered via inspiration-synchronised or continuous VMN was 10.9% lower during pressure-controlled continuous mandatory ventilation, versus a volume-controlled mode [10]. VCV has also been seen to have potential advantages over PCV in terms of greater lung deposition of radiolabelled aerosol generated with VMN (15.1% versus 10.5% with PCV) in 17 post-operative neurosurgery patients, although these individuals did not have pulmonary disease [9]. In the current study, evaluation of deposition by functional region suggests that a more stable and predictable inspiratory flow profile generated by B1 waveforms may allow deeper lung penetration, while the decelerating flow pattern characteristic of B2 (PCV-like) may limit distal transport and increase exhaled losses. The observation of similar C/P ratios with both B1 and B2 suggests that ventilator mode may have minimal impact on the relative pattern of aerosol distribution within the lung when droplet characteristics are held constant.

There is growing evidence in the literature to support the value of in silico modelling when evaluating aerosol deposition within the lung. Three-dimensional CFD has been used to simulate aerosol deposition, combining high-resolution CT scans of 20 patients with chronic obstructive pulmonary disease (COPD) and using reference gamma scintigraphy data to validate the findings [23]. In silico functional respiratory imaging in 20 patients with COPD has also been used to show differing airway deposition profiles with pMDI and dry powder inhaler (DPI) [24]. An additional comparison of airway deposition of aerosol delivered via pMDI or DPI has been performed using a three-dimensional airway model produced from 20 patients with COPD [25]. The Twinhale^®^ system described here differs from those previously presented as it is a validated, physics-based system. As such, it provides a predictive model, which contrasts with previous models that simply visualise the in vitro experiment. In addition, by utilising a patient-specific, whole-lung model, it provides a complete and realistic three-dimensional geometric representation of an individual’s lungs. This is achieved by including the full tree of conducting airways as well as alveolar tissue elements connected to the terminal airways. The mechanical submodel accounts for airflow and tissue strain in these airway segments and alveolar tissue elements, including the bidirectional coupling of flow and strain as key mechanism of breathing. In addition, the current in silico study is the first to describe variation in deposition under mechanical ventilation conditions. The computational modelling approach therefore offers a powerful tool for advancing both device and drug development by enabling high-resolution, patient-specific simulation of aerosol distribution within the lungs. This method overcomes many of the spatial and practical limitations associated with traditional imaging techniques such as planar scintigraphy, which provides only low-resolution, two-dimensional data and requires radiolabelling of aerosols. Unlike conventional methods, the in silico framework also allows direct assessment of aerosol behaviour and deposition at the site of action – information that is otherwise difficult or impossible to measure experimentally. By integrating detailed physics- and physiology-based representations of the respiratory system, this non-invasive modelling platform provides unique insight into aerosol deposition dynamics in ventilated patients. Once evaluated, scaled and appropriately validated in a range of pathological scenarios, such in silico approaches have the potential to reduce the need for invasive clinical studies and limit patient exposure to radiation, which may help to overcome some of the logistical and regulatory challenges associated with the radiolabelling of aerosolised drugs. Beyond its clinical research advantages, this approach offers potential to support the drug development process. Through simulation of the full drug delivery pathway, it could help to reduce reliance on extensive in vivo and in vitro testing, thereby lowering costs and shortening development timelines [26–28]. In principle, this in silico whole-lung model is also well suited to evaluating region-specific targeting strategies. By modifying droplet-size distribution, inspiratory flow profile, timing of aerosol delivery relative to the breath cycle, and device position in the circuit, different deposition distributions can be simulated and compared, as demonstrated in a previous in silico model [29]. Because these in silico models can be adapted to reflect patient-specific airway geometries, they can indeed be used to predict aerosol deposition patterns across a range of disease states, age groups and anatomical variations. This capability paves the way for more personalised approaches to respiratory therapy, where drug formulations and delivery devices can be optimised for individual patients. Such targeted delivery strategies could enhance treatment efficacy, minimise systemic side effects, and ultimately improve patient outcomes. Therefore, this work lays the groundwork for future multi-subject investigations aimed at validating model predictions in mechanically ventilated patients and assessing the generalizability of data across diverse patient populations and clinical scenarios. Expansion to a broader dataset will enable evaluation of intersubject variability and strengthen the translational relevance of this modelling framework.

Several limitations of this study should be acknowledged. First, Twinhale^Ⓡ^ produces deterministic outputs; for a given set of inputs the solver returns a single, reproducible deposition result without an associated confidence interval or probability distribution. This variable input comprises three principal categories: the airway geometry (derived from segmented CT imaging, which carries uncertainty, e.g., through segmentation choices), the aerosol characterisation (specified in terms of mass emission rate and droplet size distribution that may vary over time to account, for example, for dead space volume effects and time-dependent source behaviour) and the set of flow boundary conditions (defined by ventilation settings and inhalation–exhalation–breath-hold manoeuvres), all of which shape the velocity field and hence the deposition pattern.

Regarding the modelling process, the oropharyngeal curvature and ETT are not explicitly resolved in the CFD domain and aerosol losses attributable to ETT curvature and the pharyngeal region are captured in the in vitro DD measurement, which was obtained at the ETT outlet under clinically representative ventilation conditions. In addition, humidity-dependent aerosol dynamics were not explicitly modelled. The simulations used a fixed droplet-size distribution and did not resolve additional hygroscopic growth, evaporation, or condensation within the airways. This is relevant because humidity can alter aerosol size and deposition behaviour, as shown in prior work on hygroscopic aerosol growth and respiratory-tract deposition and in studies of aerosol delivery during invasive mechanical ventilation [30]. However, the droplet-size distribution used as model input was obtained from in vitro aerosol characterization at the end of the ETT, after passage through the ventilation limb, including the humidifier. Humidity-dependent changes occurring upstream of the patient interface are therefore reflected in the measured input distribution. Remaining droplet-size changes within the warm and humid lung environment are expected to be smaller, because evaporation and hygroscopic growth processes are thermodynamically fast and can approach equilibrium over short timescales. The results should therefore be interpreted as conditional on the measured droplet-size distribution at the ETT outlet. In addition, device activation timing relative to the breathing cycle may influence aerosol delivery, particularly for continuously operating nebulizers, where aerosol may be generated during expiration, and for pMDI delivery, where actuation timing relative to inspiration can affect circuit and airway losses.

Importantly, the simulations were performed using a single-subject model of a healthy lung, as here, the aim of the study was to evaluate the modelling methodology itself rather than clinical applicability. We fully acknowledge that the aerosol deposition patterns predicted for a healthy lung are not directly transferable to individuals with ARDS, COPD, pneumonia or other conditions causing heterogeneous compliance, airway obstruction or atelectasis. These pathological features would substantially alter local flow distribution, increase dead-space ventilation in consolidated regions, and likely shift deposition toward patent airways. The results of the current study therefore cannot be generalized to patient populations with differing anatomical or pathological characteristics. Indeed, even intersubject anatomical variability can substantially alter deposition behaviour. Similarly, only single representative cases were evaluated for each device category and for each flow profile. As such, the results should be interpreted as illustrative rather than definitive, reflecting proof-of-concept comparisons rather than population-level variability. Furthermore, while the model identified notable differences in predicted lung deposition across devices and the B1 and B2 flow waveforms, the clinical implications of these differences remain untested. Whether variations in regional or total aerosol deposition directly translate into improved therapeutic outcomes or patient benefit was not assessed within the scope of this work. It is also important to recognise that, despite its high-resolution and physiological basis, in silico modelling cannot fully reproduce the complexity of the in vivo environment. Factors such as inter-subject variability in airway geometry, mucus layer characteristics, lung mechanics, and device-patient interaction are challenging to capture comprehensively in a computational framework. In addition, a formal flow-rate sensitivity analysis was not performed. The observed differences between B1 and B2 arise from their distinct waveform profiles rather than a step-change in peak flow, making direct flow-rate sensitivity comparison non-trivial. In addition, the use of these standardized, idealized test waveforms means that direct comparison to clinical ICU ventilator mode studies requires caution.

In conclusion, these findings support the feasibility of using a patient-specific in silico whole-lung model to compare predicted aerosol deposition under controlled conditions. The high-resolution simulations successfully captured detailed spatial patterns of aerosol delivery and deposition, revealing fine-scale variations that reflect the complex interplay between device design and ventilation strategy. These data also highlight the promise of computational modelling as a powerful, non-invasive tool to inform aerosol therapy optimisation, guide device selection and advance the development of targeted, patient-tailored respiratory treatments. However, the results should be interpreted as mechanistic deposition data rather than evidence of clinical superiority or therapeutic benefit. While higher predicted deposition may indicate improved local drug delivery, the clinical meaning of the deposition fractions depends on the drug, required dose at the target site, dose-response relationship, therapeutic window, target lung region, disease condition, and patient-specific ventilation patterns. Further validation in multi-subject models and clinical studies will be required to determine whether these predicted deposition differences translate into meaningful clinical benefit.

## Supplementary Information

Supplementary Material 1: Supplementary Table 1. In vitro aerosol characterisation. Supplementary Fig. 1. Flow profile of B1, a sinusoidal (VCV-like) waveform and B2, a rectangular pressure (PCV-like) waveform. Supplementary Table 2. Numerical deposition values.

## Data Availability

The datasets used and/or analysed during the current study are available from the corresponding author on reasonable request.

## Abbreviations

API: Active pharmaceutical ingredient
ARDS: Acute respiratory distress syndrome
A-VMN: Aerogen vibrating mesh nebuliser
B1: Sinusoidal flow waveform
B2: Rectangular pressure-driven flow waveform
C/P: Central-to-peripheral
CFD: Computational fluid dynamics
COPD: Chronic obstructive pulmonary disease
CT: Computed tomography
DD: Delivered dose
DPI: Dry powder inhaler
ETT: Endotracheal tube
FRC: Functional residual capacity
FSI: Fluid–structure interaction
GSD: Geometric standard deviation
HMEF: Heat and moisture exchange filter
ICU: Intensive care unit
JN: Jet nebuliser
PEEP: Positive end-expiratory pressure
pMDI: Pressurised metered dose inhaler
PCV: Pressure-controlled ventilation
SPECT: Single-photon emission computed tomography
VCV: Volume-controlled ventilation
VMD: Volumetric median diameter
VMN: Vibrating mesh nebuliser
