# Supplementary material for: Evaluation of aerosol delivery in ventilated patients via vibrating mesh nebuliser versus other delivery devices using a patient-specific in silico whole-lung model
Source: Respir Res. 2026 Aug 11;27:435. doi: 10.1186/s12931-026-03865-2 (PMC13741754; doi:10.1186/s12931-026-03865-2)
Supplement: Supplementary file 1 [file 12931_2026_3865_MOESM1_ESM.docx]

**Supplementary Table 1.** *In vitro* aerosol characterisation

| **Device** | **Volumetric median diameter** | **Geometric standard deviation** | **Delivered  dose** |
| --- | --- | --- | --- |
| **Phase 1: device comparison** |  |  |  |
| A-VMN* | 2.82 μm | 1.75 | 26.99% |
| JN | 2.88 μm | 1.95 | 9.88% |
| pMDI | 2.36 μm | 1.77 | 14.23% |

*Aerosol characteristics of A-VMN were used for both B1 and B2 simulations to isolate the effect of the ventilation flow profile

A-VMN, Aerogen vibrating mesh nebuliser; JN, jet nebuliser; pMDI, pressurised metered dose inhaler

**Supplementary Fig. 1. Flow profile of B1, a sinusoidal (VCV-like) waveform and B2, a rectangular pressure (PCV-like) waveform**

PCV, pressure-controlled ventilation; VCV, volume-controlled ventilation

**Supplementary Table 2. Numerical deposition values**

| **A. Device comparison** | | | | | |
| --- | --- | --- | --- | --- | --- |
| **Region / metric (% or ratio)** | **A-VMN** | | **JN** | | **pMDI** |
| Right upper lobe deposition | 3.5 | | 1.8 | | 1.8 |
| Right middle lobe deposition | 1.8 | | 1.0 | | 1.0 |
| Right lower lobe deposition | 7.4 | | 3.5 | | 4.5 |
| Left upper lobe deposition | 4.5 | | 1.6 | | 2.4 |
| Left lower lobe deposition | 6.0 | | 2.3 | | 3.2 |
| Left lung central deposition | 0.8 | | 0.4 | | 0.4 |
| Left lung peripheral deposition | 9.8 | | 3.4 | | 5.3 |
| Right lung central deposition | 1.1 | | 0.5 | | 0.5 |
| Right lung peripheral deposition | 11.7 | | 4.7 | | 6.2 |
| Thoracic airway deposition | 5.3 | | 2.3 | | 2.1 |
| Alveolar tissue deposition | 18.8 | | 6.5 | | 10.6 |
| Exhaled fraction | 2.8 | | 1.0 | | 1.5 |
| Left lung C/P ratio | 0.08 | | 0.11 | | 0.07 |
| Right lung C/P ratio | 0.09 | | 0.11 | | 0.09 |
| **B. Flow profile comparison** | | | | | |
| **Region / metric (% or ratio)** | | **B1** | | **B2** | |
| Right upper lobe deposition | | 3.6 | | 2.3 | |
| Right middle lobe deposition | | 1.8 | | 1.4 | |
| Right lower lobe deposition | | 7.4 | | 4.9 | |
| Left upper lobe deposition | | 4.5 | | 3.2 | |
| Left lower lobe deposition | | 6.0 | | 4.0 | |
| Left lung central deposition | | 0.8 | | 0.5 | |
| Left lung peripheral deposition | | 9.8 | | 6.7 | |
| Right lung central deposition | | 1.1 | | 0.8 | |
| Right lung peripheral deposition | | 11.7 | | 7.9 | |
| Thoracic airway deposition | | 5.3 | | 4.1 | |
| Alveolar tissue deposition | | 18.8 | | 12.1 | |
| Exhaled fraction | | 2.8 | | 10.4 | |
| Left lung C/P ratio | | 0.08 | | 0.08 | |
| Right lung C/P ratio | | 0.09 | | 0.10 | |

A-VMN, Aerogen vibrating mesh nebuliser; B1, sinusoidal flow waveform; B2, rectangular pressure-driven flow waveform; C/P, central-to-peripheral; JN, jet nebuliser; pMDI, pressurised metered dose inhaler
